# Using tools with real and imagined tool movements

**DOI:** 10.3389/fpsyg.2014.00515

**Published:** 2014-06-17

**Authors:** Jochen Müsseler, Peter Wühr, Michael Ziessler

**Affiliations:** ^1^Work and Cognitive Psychology, RWTH Aachen UniversityAachen, Germany; ^2^Technical University of DortmundDortmund, Germany; ^3^Liverpool Hope UniversityLiverpool, UK

**Keywords:** tool use, sensorimotor transformation, imagery, imagination, stimulus-response compatibility, action effect, ideomotor theory

## Abstract

When using lever tools, subjects have to deal with two, not necessarily concordant effects of their motor behavior: the body-related proximal effects, like tactile sensations from the moving hand, and/or more external distal effects, like the moving effect points of the lever. As a consequence, spatial compatibility relationships between stimulus (S; at which the effect points of the lever aim at), responding hand (R) and effect point of the lever (E) play a critical role in response generation. In the present study we examine whether the occurrence of compatibility effects needs real tool movements or whether a similar response pattern can be already evoked by pure mental imaginations of the tool effects. In general, response times and errors observed with real and imagined tool movements showed a similar pattern of results, but there were also differences. With incompatible relationships and thus more difficult tasks, response times were reduced with imagined tool movements than compared with real tool movements. On the contrary, with compatible relationships and thus high overlap between proximal and distal action effects, response times were increased with imagined tool movements. Results are only in parts consistent with the ideomotor theory of motor control.

## INTRODUCTION

Responding to a stimulus is faster and more accurate when stimulus location and response location spatially corresponds than when they do not. This effect is well known as spatial stimulus-response compatibility (SR compatibility). Explanations of SR compatibility often assume that the presentation of a stimulus activates automatically the ipsilateral response. This activation is advantageous in spatially corresponding conditions, but results in a response conflict in spatially non-corresponding conditions. The solution of the response conflict increases the time needed to select the response and the probability of selecting the wrong response (for an overview see Proctor and Vu, 2006).

Furthermore, in the last two decades studies demonstrated that response times and errors in spatial compatibility tasks are not only determined by the spatial relationship between stimulus and response, but also by the location of the intended action effects (e.g., Hommel, 1993; Kunde, 2001). The theoretical background of these studies was that actors select, initiate, and execute a movement by anticipating the movement’s sensory effects (ideomotor principle, see, e.g., Hommel et al., 2001a,b; Shin et al., 2010). These may be representations of body-related effects, like tactile sensations from the moving finger, and/or representations of more external effects, like the illuminating bulb when the switch is turned on.

However, when considering the chain “Stimulus → Response → Effect,” different compatibility relationships come into play. Beside the mentioned SR compatibility, performance might be also influenced by the spatial relationships between stimulus and action effect (SE compatibility) and/or by the spatial relationships between response and action effect (RE compatibility). Investigating the use of lever tools where the moving effect points of a lever represent the (anticipated) action effect, proofed to be an easy way to decouple – at least in parts – the different compatibility relationships (e.g., Kunde et al., 2007; Massen and Prinz, 2007; Müsseler et al., 2008; Beisert et al., 2010; Massen and Sattler, 2010). In these studies the SR relationship is the correspondence (or non-correspondence) between stimulus location and hand-response direction. The SE relationship is the correspondence (or non-correspondence) between stimulus location and the direction of the lever’s effect point. Thus, a compatible SE relationship represents the situation in which the lever’s effect points have to reach at the stimulus and an incompatible SE relationship represents the situation in which the effect points have to be shifted away from it. Finally, the RE relationship reflects the correspondence (or non-correspondence) between hand-response direction and the direction of the spatial effect point of the lever. With a compatible RE relationship, the hand and the lever’s effect points move in the same direction, while with an incompatible RE relationship the hand and the lever’s effect points move in the opposite direction. Thus, an incompatible RE relationship requires an inverse tool transformation.

**FIGURE 1:**
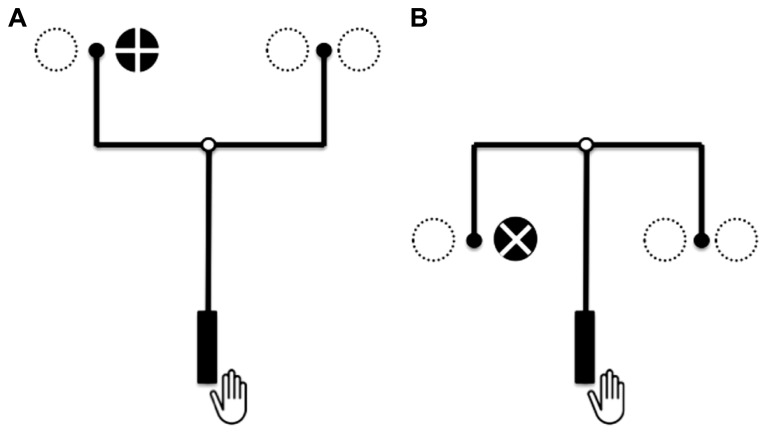
**The U-lever (A) and the inverted U-lever (B)** in the study of Müsseler and Skottke (2011). Imperative stimuli were “+” or “ × ” indicating the requirement to move the lever’s effect point toward the stimulus or to move the lever’s effect point away from it. The open circles in the middle of the cross bar represent the pivot, the filled circles at the ends of the upward or downward rods the lever’s effect points. The larger dotted circles indicated other stimulus positions.

To our knowledge, SR, SE, and RE compatibilities were varied simultaneously only in a study by Müsseler and Skottke (2011). In their experiment the authors used an U-lever and an inverted U-lever with a pivot: the tool consisted of a vertical rod with a grip at the bottom part and a centrally placed crossbar in the upper part (**Figure 1**). The pivot point was in the middle of the horizontal rod and the tool’s effect points were at the ends of additional upward or downward oriented rods attached to the crossbar. Using these tools made it possible to manipulate SR, SE, and RE compatibilities independently of each other in a full 2 × 2 × 2 design allowing to examine the contribution of each compatibility relationship and their interactions to response times and errors^1^.

The main outcome of this study was that response times and errors were drastically increased with an inverse tool transformation, that is when hand movement and the lever’s effect point move in opposite direction (incompatible RE relationship see also Kunde et al., 2007; Müsseler et al., 2008; Massen and Sattler, 2010). For instance, in **Figure 1A**, a hand movement to the left results in an effect point movement to the right. This situation is disadvantaged compared with the situation when hand movement and the lever’s effect points move in the same direction (compatible RE relationship; **Figure 1B**). Additionally, it turned out to be easier to reach with the levers’ effect points at the stimulus (compatible SE response) than to shift the effect points to the contrary side (incompatible SE response). However, at least this finding has to be interpreted with the significant SE–RE interaction and the significant three-way SR-SE-RE interaction. In short, the interactions came about by substantial differences within the compatible RE conditions, while only minor differences were observed within incompatible RE conditions.

**FIGURE 2:**
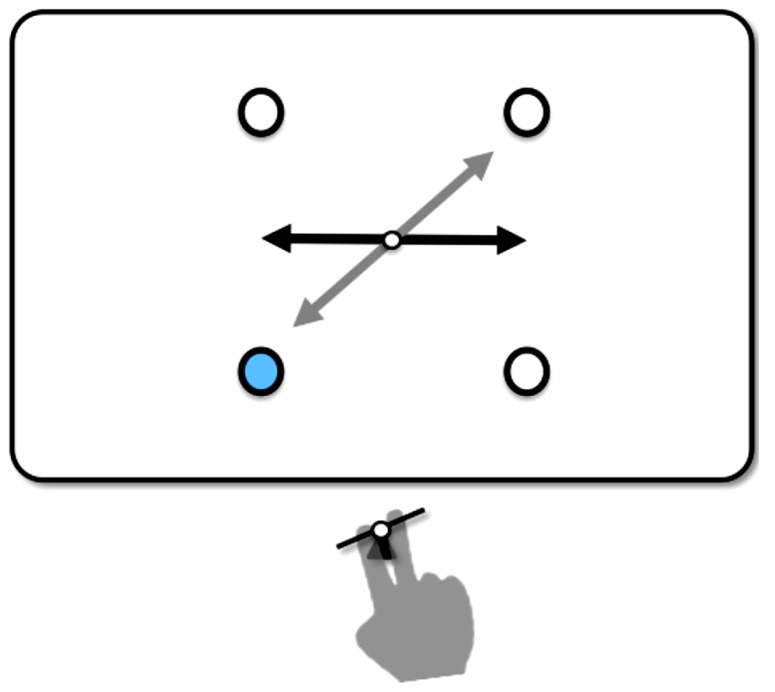
**Schematic illustration of the experimental setup.** In response to a color change (here light blue) of one of the four disks, participants operated a rocker switch with the index finger and middle finger. With the key-press, the rocker on the display (horizontal straight line with arrows at both ends) moved from the horizontal home position to the end position (gray line, here in correspondence with the hand movement). The figure is not to scale.

The aim of the present study was twofold. The first aim was to replicate the findings of Müsseler and Skottke (2011) with a simpler tool. One objection against the U-shaped and inverted U-shaped lever is that hand movements and tool-effect movements are only indirectly coupled through the pivot point. Therefore, in the present experiment participants operated with the index finger and middle finger a rocker switch. With a key-press on the rocker switch, a rocker presented on a display moved in direct correspondence (or non-correspondence) to the hand movement (see **Figure 2**). The rocker was the participant’s tool for pointing to the imperative stimuli.

**FIGURE 3:**
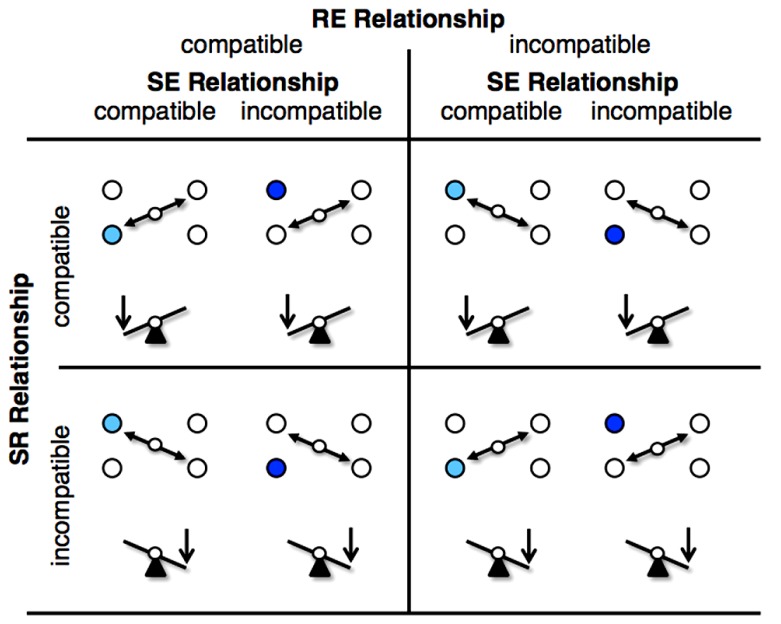
**SR, RE, and SE relationships with the rocker.** In the figure the imperative light blue disk indicated to move the nearest rocker’s effect point toward the stimulus (compatible SE relationships) and the dark blue disk indicated to move the rocker’s effect point away it (incompatible SE relationships). Further, the rocker on the display moved in correspondence with the hand movement (compatible RE relationships) or in non-correspondence (incompatible RE relationships, which indicated an inverse tool transformation). Finally, the key-press on the rocker switch corresponded spatially with the side of disk presentation (compatible SR relationships) or did not correspond spatially (incompatible SR relationships). In the figure, only left stimulus presentations are depicted, right stimulus presentations were varied correspondingly. Circles represent pivot points, vertical arrows the side of the key-press.

**Figure 3** illustrates the various SR, RE, and SE relationships with the rocker. A compatible SR relationship was present when the side of the key-press corresponded with the side of disk presentation, otherwise it was SR incompatible. An imperative light blue disk indicated to move the nearest rocker’s effect point toward the stimulus, exposing the compatible SE relationships. A dark blue disk indicated to move the rocker’s effect point away from the stimulus and thus represent an incompatible SE relationships. Further, the rocker on the display moved in correspondence with the hand movement, which resembles a compatible RE relationship. A non-correspondence between rocker movement and hand movement notify an incompatible RE relationships, which agreed with the inverse tool transformation^2^.

The second aim of our study was to contrast real vs. imagined rocker movements, that is, participants were asked to operate the rocker switch with corresponding movements of the rocker and without such movements displayed on the screen. In the latter condition, they should only imagine that the rocker (the tool for the pointing movements) moved.

Imagined tool movements are often studied in the context of mental training demonstrating generally an improvement in performance through previous imaginations [e.g., playing tennis with a tennis racket (Noel, 1980) or playing golf using a golf club (Taylor and Shaw, 2002)]. By comparing real with imagined movement times, other studies showed, for instance, that mental models of the tool mechanics are used during imagery (Schwartz and Holton, 2000), that the thickness of a painting tool and thus characteristics of the tools’ effect influence imagery time (Rieger and Massen, 2014), or that the speed-accuracy relationships of Fitts’ law are present in real and imagined tool use (Macuga et al., 2012). Consequently, movements with real and imagined tool use are assumed to recruit similar processing mechanisms (see also Jeannerod, 1994, 2001; Davidson and Wolpert, 2005; Higuchi et al., 2007; Munzert et al., 2009).

However, the movements investigated in previous studies of imagined tool movements are relatively long lasting and complex. They also often require closed-loop control when performed under real conditions. Consequently, the observed effect of imagery can depend on many factors and it remains open to what extent they include the imaginations of tool transformations as part of the planning of the complex action. In contrast, only short ballistic, open-loop movements are necessary to press the rocker switch in the present experiment. Moreover, as we used a computer-animated version of a lever^3^, the lever “moves” in direct response to the key-press within one vertical retrace of the monitor to its end position. In other words, the responses are fully preplanned and executed before the tool-effect movements actually take place. Consequently, compatibility effects should be fully visible in response time differences, independently of whether a rocker movement occurred in reality or in imagination.

Another motivation for comparing conditions with real and imagined tool movements arises from ideomotor theory. With regard to the ideomotor principle, tool movements are initiated by both the anticipation of the body-related kinesthetic effects and the anticipation of the tool effects in the environment. Whenever a tool is used, actors’ intentions are usually directed to the tool’s effect points. We have already pointed out that especially when feature overlap between hand movement and tool movement is high, actors are less aware of their own hand movements and the distal tool effects become predominant (Sutter et al., 2013; for empirical evidence see, e.g., Rieger et al., 2005; Müsseler and Sutter, 2009; Sülzenbrück and Heuer, 2009). In other words, with compatible RE relationships, the information processor seems to work in an automated manner with the tool effects. If the anticipated distal tool effects are used to initiate the key-press response, as is assumed by the ideomotor account, key-press times should not vary between conditions of real and imagined tool movements, especially under compatible conditions.

However, with incompatible relationships in the chain “Stimulus → Response → Effect,” the situation might be different. If feature overlap between hand movement and tool movement is low and if a transformation between them is obvious, proximal action effects might interfere with the distal tool actions. The incompatible relationship requires a tool transformation and consequently the actors might perceive the task as more difficult (cf. Sutter et al., 2013). In this case, it might be easier for the actor to apply SR-translation rules to the task and thus to ignore the distal tool movements. If so, in conditions with real tool movements, the anticipation of the discordant tool effects might hamper response execution, while in conditions with imagined tool movements, discordant tool effects are easier to “ignore.” Therefore, we expect increased response times with real tool movements especially under incompatible conditions.

## MATERIALS AND METHODS

### PARTICIPANTS

Twelve students (10 female, between 19 and 31 years of age, mean age 22.3 years) from RWTH Aachen University participated in the experiment for pay or course credit.

### APPARATUS AND STIMULI

The experiment was carried out in a dimly lit chamber and was controlled by an Apple Macintosh computer with Matlab software (Mathworks) using the Psychtoolbox-3 extension (Kleiner et al., 2007). Participants responded with their index or middle finger of their preferred hand by pressing down the left or right side of a rocker switch, which could release a corresponding or non-corresponding movement of the rocker displayed on a 22” color CRT monitor (Iiyama Vision Master Pro 513, 100-Hz refresh rate, 1024 × 768 pixel). The rocker was presented at screen center and consisted of a black straight line (200 pixel) with arrows at both ends and a pivot (8 pixel) in the middle (cf. **Figure 2**).

A dark blue or light blue disk was displayed in one of four gray disks (each with a diameter of 40 pixel), which formed a virtual square (240 × 240 pixel) surrounding the rocker. The participant’s head was placed on a chin rest 500 mm in front of the monitor. The blue disks served as imperative stimuli, the gray disks were placeholders for possible stimulus positions.

### DESIGN

The experiment had a 2 (real vs. imagined tool movement) × 2 (SR compatible vs. incompatible) × 2 (RE compatible vs. incompatible) × 2 (SE compatible vs. incompatible) repeated measurement design. The four combinations of the factors “real vs. imagined tool movement” and “RE compatibility” were presented block-wise at two different days with the sequence of blocks balanced between participants. The only restriction was that the conditions of real tool movements and imagined tool movements were presented consecutively at 1 day. For example, at the first day a participant performed the RE-compatible trials with the real tool movements and then with the imagined tool movements (or vice versa). At least 1 week later, the participant performed the RE-incompatible trials with the real tool movements and then with the imagined tool movements (or vice versa). Within these blocks, all combinations of SR and SE compatibility were presented in a randomized order.

Altogether, participants worked through a total of 960 trials. The first blocks were considered as practice trials and were not analyzed. Thus, each cell of the design was filled with 50 repeated-measurement trials. Dependent measures were median response times and the error percentages of each participant.

### PROCEDURE

Participants were instructed in written form. They were informed that a left or right key-press on the rocker switch produced a corresponding (RE compatible) or a reversed turn (RE incompatible) of the rocker on the screen. In conditions of imagined tool use, participants were asked to imagine the corresponding tool movement only.

The experiment started with the presentation of the four gray disks and the rocker, which remained visible until the end of the experiment. At the beginning of each trial, the rocker was in the horizontal home position. When one of the four gray disks changed its color to light or dark blue, participants were required to press the right or left side of the rocker switch as fast and accurately as possible. The light blue disk indicated to move the nearer effect point of the rocker (the left or right arrow) toward the stimulus and the dark blue disk indicated to move the nearer effect point away from the stimulus. The key-press immediately caused a corresponding or non-corresponding shift of the rocker to the end position with the next vertical retrace of the monitor. Through the phi phenomenon observers perceived a movement of the rocker between the home position and the end position. The rocker turned back to the home position after the release of the key. The next trial started after 1.5 s.

An error feedback was given, if participants had made the wrong response (a tone of 440 Hz with a duration of 50 ms) or if response times were lower than 100 ms or exceeded 2,000 ms (a tone of 880 Hz with a duration of 50 ms). At each day, the experiment lasted about 30 min including short breaks every 40 trials.

## RESULTS

**FIGURE 4:**
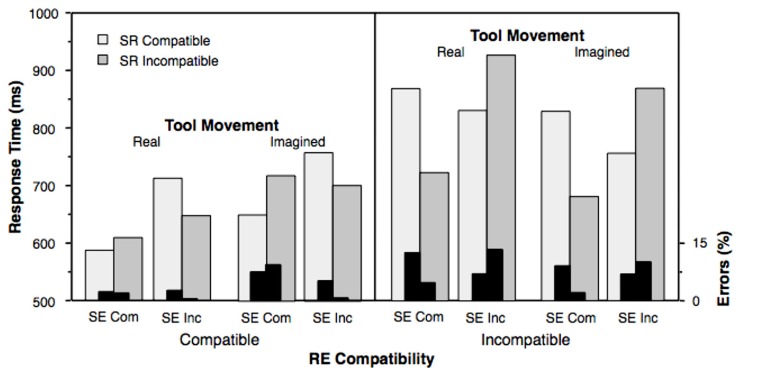
**Mean response times (light and dark gray bars, left *y*-axis) and error percentage (black bars, right *y*-axis)** of the stimulus-response (SR), response-effect (RE), and stimulus-effect (SE) relationships in dependence of real and imagined tool movements. Com, compatible; Inc, incompatible.

Median response times and percentage of errors of each participant were entered into 2 (real vs. imagined tool movement) × 2 (SR compatible vs. incompatible) × 2 (RE compatible vs. incompatible) × 2 (SE compatible vs. incompatible) analysis of variance (ANOVA) with repeated measurements. Results are shown in **Figure 4**. We first focus on the results of SR, RE, and SE compatibility and their interactions averaged over real and imagined tool movements. After that we look at this factor and its possible interactions with the compatibility conditions.

A significant RE effect was observed in the reaction-time analysis, *F*(1,11) = 23.09, *p* = 0.001, $\eta_{p}^{2}$ = 0.677, and in the error analysis, *F*(1,11) = 12.99, *p* = 0.004, $\eta_{p}^{2}$ = 0.541. Responses under compatible RE relationships were performed 138 ms faster and with 4.6% less errors than under incompatible RE relationships (673 vs. 811 ms and 3.7 vs. 8.3%). In other words, when the rocker movement on the display was in correspondence with the hand movement on the rocker switch (left panel of **Figure 4**), response times and errors decreased compared with a non-correspondence of hand and rocker movements (right panel of **Figure 4**).

Other significant main effects were that responses were performed faster (708 ms) under compatible SE relations than incompatible SE relations (775 ms), *F*(1,11) = 29.14, *p* < 0.001, $\eta_{p}^{2}$ = 0.726, and that errors in SR compatible trials were significantly increased as compared to errors in SR incompatible trials (6.6 vs. 5.3%), *F*(1,11) = 10.63, *p* = 0.008, $\eta_{p}^{2}$ = 0.491. However, these findings have to be qualified by significant interactions. The two-way interaction between SE and SR compatibility was significant in the response time analysis, *F*(1,11) = 7.78, *p* = 0.018, $\eta_{p}^{2}$ = 0.414, and in the error analysis, *F*(1,11) = 8.04, *p* = 0.016, $\eta_{p}^{2}$ = 0.422. Furthermore, the three-way interaction between SE, SR, and RE compatibility was significant in the response time analysis, *F*(1,11) = 25.81, *p* < 0.001, $\eta_{p}^{2}$ = 0.701, and in the error analysis, *F*(1,11) = 29.04, *p* < 0.001, $\eta_{p}^{2}$ = 0.725. The three-way interaction reflects the finding that under compatible RE relations (**Figure 4**, left panel) responses in SR compatible trials were advantaged if the SE relationship was compatible, but disadvantaged if the SE relationship was incompatible. Under incompatible RE relations (**Figure 4**, right panel) this pattern of results was reversed. However, there is also a possibly more simple description of those results, if the left–right SR compatibility is replaced by the compatibility between the stimulus position and the direction of the required response. A closer inspection of the three-way interaction revealed that responses were faster and less error prone when the stimuli appeared in the lower position as compared to their appearance in the upper position (697 vs. 786 ms, 4.0 vs. 8.0%; cf. **Figures 3** and **4**). Corresponding *post hoc t*-tests were significant for response times with *t*(11) = 5.08, *p* < 0.001 and for errors with *t*(11) = 5.39, *p* < 0.001, two-tailed.

**FIGURE 5:**
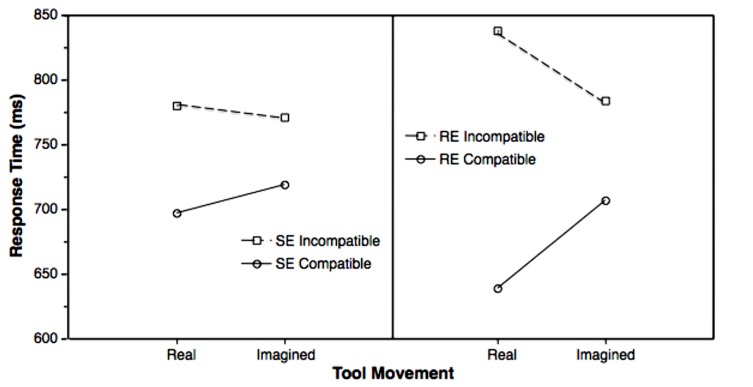
**Interactions of SE compatibility (left panel) and RE compatibility (right panel) with real vs. imagined tool movements**.

When comparing response times and errors with regard to real and imagined tool movements, the findings appear amazingly consistent. However, two differences showed up as interactions in the reaction-time analysis. First, the SE compatibility effect was larger with real tool movements (difference = 83 ms) than with imagined tool movements (difference = 52 ms; cf. **Figure 5**, left panel), producing a significant interaction between “SE compatibility” and “real vs. imagined tool movement,” *F*(1,11) = 8.74, *p* = 0.013, $\eta_{p}^{2}$ = 0.443.

Second, there was a tendency toward an interaction of “RE compatibility” and “real vs. imagined tool movement,” *F*(1,11) = 4.18, *p* = 0.066, $\eta_{p}^{2}$ = 0.275 (cf. **Figure 5**, right panel). This result indicated that also the RE compatibility effect was larger with real tool movements (difference = 199 ms) than with imagined tool movements (difference = 77 ms; cf. **Figure 5**, right panel). Other effects were not significant, also not in the error analysis.

## GENERAL DISCUSSION

In a reaction-time experiment the spatial compatibility relations between stimulus and response location, between stimulus and SE and between response and RE were varied independently of each other. In addition, in half of the trials the response-effect was actually visible whereas in the other half of trials the participants only imagined the effect. As we did in the results section, we will first discuss the results of SR, RE, and SE compatibility and their interactions.

The analyses of the response times and errors indicate that all types of compatibility relationships were involved in the planning and execution of the responses. However, the strength of the compatibility effects varied between the different relationships. The most prominent compatibility effects were observed for RE compatibility. Under all conditions and independent on the other two compatibility relationships participants needed more time if the movements of the fingers on the rocker switch were incompatible to the movements of the rocker on the screen (see also Kunde et al., 2007; Müsseler et al., 2008; Massen and Sattler, 2010). Under compatible conditions, participants can plan the responses using the same features than those used for the anticipation of the intended effect. However, if the RE relations are incompatible, an additional transformation process is necessary that translates the features of the intended effect into the features of the response to achieve this effect. In line with the ideomotor principle, this underlines the importance of the response-effects for the control of the responses. However, the result also shows that the assumption of the ideomotor principle that the responses are directly activated by the anticipation of their effects might be too simple. To activate a response, the effect features need to be translated into response features and this translation process is facilitated under compatible conditions, i.e., if there is an overlap between features of the effects, the stimuli and the responses.

Compared to the RE compatibility, the effects of SE compatibility were on average about 50% smaller and depended on the other compatibility relationships. Only if the RE and SR relations were both compatible or both incompatible, an SE compatibility effect was observed. Similarly, SR compatibility effects were only observed, if RE and SE relations were both compatible or both incompatible. It should be noted that SR compatibility effects were smaller than the SE compatibility effects and for two conditions the SR compatibility effects were negative. If the RE relations were compatible and the SE relations incompatible, compatible SR relations were associated with longer response times than incompatible SR relations. The same inverted compatibility effect was found if the RE relations were incompatible and the SE relations compatible (see also Müsseler et al., 2008, for a similar observation). In other words, the control of the responses is dominated by the compatibility between features of the responses and the effects and between features of the stimuli and the effects. Within the complex pattern of compatibility relations, SR compatibility seems to play only a minor role. It might be that the participants rarely use the left–right location of the stimuli for the coding of the responses because the location of the stimulus does not allow a decision on the response. Instead they could use configurative features. For example, under the RE compatible condition, if a dark blue stimulus appears on the main diagonal of the screen (upper left or lower right stimulus position) a right response would be required. Possible SR compatibility effects are overwritten by other compatibility relations. As we have shown in the results section, if the stimuli appeared in the lower position responses were faster and more accurate. Because all four responses consisted in downward responses, for stimuli in the lower position the stimulus position and the movement direction were compatible whereas this relationship was incompatible for stimuli in the upper position. However, it is also worth to note that the effects of the lower and upper positions on response times and errors were probably evoked by the tool, that is by the rocker switch. Müsseler et al. (2008, Experiment 2, Figure 4) observed in a comparable setup without a tool only minor effects of a few milliseconds at upper and lower positions, but strong effects as in the present experiment when the tool was presented.

In sum, when considering the results with regard to the SR, SE, and RE compatibility, the present findings replicated successfully the study of Müsseler and Skottke (2011) with a simpler lever tool. The observed main effects of SE and RE compatibility as well as the interactions were found in both studies and demonstrate the robustness of the results with lever tools (see also Kunde et al., 2007; Müsseler et al., 2008; Massen and Sattler, 2010). The only obvious difference seems to be that the differences within the incompatible RE conditions were more pronounced in the present study than in the previous study of Müsseler and Skottke (2011).

If the participants only imagined the movements of the rocker on the screen a similar pattern of compatibility effects emerged. This is further evidence that real and imagined movements might recruit similar processing mechanisms (cf. Jeannerod, 1994, 2001; Davidson and Wolpert, 2005; Higuchi et al., 2007; Munzert et al., 2009; Macuga et al., 2012; Rieger and Massen, 2014).

However, we also observed a significant interaction between factors SE compatibility and the tool movement (real vs. imagined) and a trend toward an interaction between RE compatibility and tool movement. If the tool movement was present on the screen as response-effect, both compatibility effects involving the tool movement (i.e., the distal effect) were more pronounced as compared to imagined tool movements. The physical absence of the tool movements reduced both the facilitating effect of compatible relationships and the inhibiting effect of incompatible relationships (**Figure 5**). On the one hand, there is evidence that the participants still use the tool effects for the control of their responses as assumed by the ideomotor principle, even if the effects are only imagined. On the other hand, the reduction of the SE and RE compatibility effects suggests that the participants rely less on the effects in controlling their responses if the tool movements are not real. The latter is difficult to explain in the theoretical framework of the ideomotor principle. If the effects were necessary for the selection of the responses, it should not matter whether the effects were physically present or only imagined. In both cases the effects have to be anticipated and the anticipated effects should then activate the response. Thus, the pattern of the compatibility effects indicates that the imagined tool movements were involved in the control of the responses, but the reduced size of the compatibility effects is also evidence that the selection of the responses does not fully depend on the anticipation of the effects.

This leads to the interesting question of what is the function of effect anticipation in the control of motor responses. As it seems from the present results, the central assumption of the ideomotor principle that effects are used for response selection is too narrow. In motor control, effects are also involved in the monitoring of the responses and the evaluation of the executed responses (e.g., Schmidt, 1975). Both functions include that the effects of a selected response are anticipated as part of response planning (Nikolaev et al., 2008; Ziessler and Nattkemper, 2011; Ziessler et al., 2012). If effects are anticipated depending on a selected response, it becomes possible to perform an internal test of the selected response: the response can be executed if the anticipated effect is in accordance with the intended effect.

Taking this idea into account, there is an alternative interpretation for the observed compatibility effects. The participants might select their responses via simple stimulus-response transformation rules (e.g., under RE compatible conditions: dark blue stimulus on the main diagonal [upper left or bottom right] → press left key, light blue stimulus on antidiagonal [upper right or bottom left] → press left key etc.). The anticipation of the response-effects, i.e., the anticipation of the rocker movement, depends on SE and RE compatibility. The faster participants get the confirmation from the internal test that the planned response will generate the intended effect the earlier the response will be executed. Under compatible conditions this procedure will facilitate the responses, under incompatible conditions the procedure might hold up the responses.

The described interpretation applies in particular to the condition in which the movement of the rocker was physically present. Under the condition of imagined rocker movements the participants learned that there was no distal tool effect of their responses. It has been shown in other experiments that participants stop to anticipate learned response-effects if the effects were removed from the experimental setting (Ziessler et al., 2012). Consequently, under the imaging condition there was no reason for the participants to anticipate non-existing effects during the planning of the responses. This could have led to the leveling of the response times. Effect related compatibility effects are still there as long as the participants follow the instruction to imagine the effects. But the compatibility effects become weaker because the anticipation of rocker movements is in conflict with the actual effects.

In conclusion, the present experiment underlines again that the anticipation of effects is an important component of response planning. This includes distal effects that are generated by tools. The function of effect anticipation does not seem to be limited to response selection. Anticipation of effects for selected responses also constitutes an internal test if the selected response will generate the intended effect. For the planning of a response including the anticipation of its effects the cognitive system uses very flexibly all existing relationships between the stimuli, the responses and the effects. The impact of distal response-effects on response planning diminishes if the effects are removed from the setting. Under theoretical aspects, that means the ideomotor principle needs at least to be amended to provide an explanation for the multiple ways of response selection and response preparation.

## Conflict of Interest Statement

The authors declare that the research was conducted in the absence of any commercial or financial relationships that could be construed as a potential conflict of interest.

## Footnotes

1 In the present study SR, SE, and RE compatibilities were also varied independently of each other with another, more simpler tool. In **Figure 3**, the concrete implementations of the compatibility relationships were explicated for the new tool, which have been also applied to the U-lever and inverted U-lever (see Müsseler and Skottke, 2011, Figure 4).

2 Analyzing the SR settings without the rocker revealed further possible relationships between stimuli and responses. Beside the spatial SR relationship, left–right responses could be differently influenced by the upper–bottom arrangement and/or by the color of the stimuli. Several findings indicate that responding to an upper stimulus is somewhat faster and more accurate with a right response than when with a left response and vice versa (orthogonal compatibility effects, for an overview see Proctor and Vu, 2006, Chap. 8). However, in the present design orthogonal compatibility effects cancel each other out. For example, an upper left light blue disk required a right response (which should be facilitated by the orthogonal relationship), but an upper right light blue disk required a left response (which should be hampered by the orthogonal relationship). Only the average of both conditions entered into the analyses. Additionally, we see no reason to assume different effects of light and dark blue stimuli on left–right responses.

3 Note, that comparing response times with a real and a computer-animated lever have shown amazingly consistent results [cf. the results of Kunde et al. (2007, Experiment 1) with a real lever and Müsseler et al. (2008, Experiment 2) with a comparable computer-animated lever; see also Müsseler and Skottke, 2011; for a direct comparison of both types of levers see Müsseler et al., 2008, Experiments 1a and 1b].
